# Understanding an evolving pandemic: An analysis of the clinical time delay distributions of COVID-19 in the United Kingdom

**DOI:** 10.1371/journal.pone.0257978

**Published:** 2021-10-20

**Authors:** Thomas Ward, Alexander Johnsen

**Affiliations:** 1 Public Health England, London, United Kingdom; 2 Joint Biosecurity Centre, London, United Kingdom; 3 Department of Health and Social Care, London, United Kingdom; Post Graduate Institute of Medical Education and Research, INDIA

## Abstract

Understanding and monitoring the epidemiological time delay dynamics of SARS-CoV-2 infection provides insights that are key to discerning changes in the phenotype of the virus, the demographics impacted, the efficacy of treatment, and the ability of the health service to manage large volumes of patients. This paper analyses how the pandemic has evolved in the United Kingdom through the temporal changes to the epidemiological time delay distributions for clinical outcomes. Using the most complete clinical data presently available, we have analysed, through a doubly interval censored Bayesian modelling approach, the time from infection to a clinical outcome. Across the pandemic, for the periods that were defined as epidemiologically distinct, the modelled mean ranges from 8.0 to 9.7 days for infection to hospitalisation, 10.3 to 15.0 days for hospitalisation to death, and 17.4 to 24.7 days for infection to death. The time delay from infection to hospitalisation has increased since the first wave of the pandemic. A marked decrease was observed in the time from hospitalisation to death and infection to death at times of high incidence when hospitals and ICUs were under the most pressure. There is a clear relationship between age groups that is indicative of the youngest and oldest demographics having the shortest time delay distributions before a clinical outcome. A statistically significant difference was found between genders for the time delay from infection to hospitalisation, which was not found for hospitalisation to death. The results by age group indicate that younger demographics that require clinical intervention for SARS-CoV-2 infection are more likely to require earlier hospitalisation that leads to a shorter time to death, which is suggestive of the largely more vulnerable nature of these individuals that succumb to infection. The distinction found between genders for exposure to hospitalisation is revealing of gender healthcare seeking behaviours.

## Introduction

The COVID-19 pandemic has had an unprecedented impact on the global population. In the United Kingdom, as of 24 February 2021, 4194785 cases have been observed [1] causing 440 369 hospitalisations and 140062 deaths, which has placed extraordinary pressure on the healthcare system. The changing landscape of COVID-19 prevalence due to non-pharmaceutical interventions (NPIs) has led to a variable aetiological clinical impact. Moreover, since the onset of the pandemic, the virus has had varying temporal consequences for different demographics, affecting the time delay parameters, which was particularly pronounced with the March 2020 outbreak in care homes [2]. Understanding these temporal time delay dynamics of infection is key for the calculation of the infection hospitalisation rate (IHR) and infection fatality rate (IFR). This in turn has implications for the accurate modelling of the pandemic and formulation of effective public health policy. For instance, the changes to the time delay dynamics are central to estimating the incubation and illness period, which is essential for defining accurate quarantine periods for those that have been infected or exposed by a contact. Tracking the phenotypic changes in the virus is now becoming more relevant due to the extent of antigenic drift observed in SARS-CoV-2 [3] and worrying mutations [4] that may have an impact upon vaccine effectiveness.

There is limited contemporary research that looks at infection to clinical outcomes and nothing we have found for this study that addresses the temporal changes or looks in detail at the distinctions between gender or by age. Much of the literature that seeks to estimate the time delay dynamics [5] has been focused on the outbreak in Wuhan, China seen in 2019 and at the start of 2020. From this period Linton et al. (2020) [6] calculated the mean time from infection to hospitalisation: 9.7 days (95% CI: 5.4, 17.0), hospitalisation to death: 13.0 days (95% CI: 8.7, 20.9), and infection to death: 20.2 days (95% CI: 15.1, 29.5). However, these estimates are predominantly from small samples and, due to the pandemic nature of this outbreak, are dependent upon the demographic structure, the quality of the healthcare system, and the epidemiological context in which they were collected.

The time between infection and a clinical outcome for infectious diseases is not precisely observed and therefore is often ‘coarsely’ recorded, that is, we observe a subset of the sample space in which the true but unobservable data actually lie [7]. Therefore, modelling of this type of data needs to adjust for its imprecise nature or it is likely that the estimates will not accurately capture the maximum likelihood or the tails of the distribution, which can be important to inform key elements of public health policy. McAloon et al. [5] found, in a meta-analysis of studies published on the incubation period of COVID-19, that this has been overlooked in much of the current literature. In this study, we employed a doubly interval censored modelling approach [8] that seeks to capture all the available information of the clinical time delay distribution.

The time delay from infection to a clinical outcome has changed in response to the evolution of intrinsic and extrinsic factors across the geography of the United Kingdom. Using the most complete clinical data presently available, we have calculated, across distinct epidemiological periods in the pandemic, the difference in the time delay distributions for hospitalisations and deaths. These periods were defined by identifying temporally unique periods that were found to be strongly associated with changes in the prevalence of SARS-CoV-2. We have further modelled the difference between age groups and by gender to understand and analyse distinctions between demographic groups.

## Methods

### Epidemiological data

Two Public Health England datasets were used in this study: the mortality line list and the Severe Acute Respiratory Infection Watch (SARI) line list [9]. The data used ranges from 1 January 2020 to 20 January 2021. The key dates used to develop the models were of symptom onset, hospitalisation, and mortality in order to measure three quantities of interest: the time from infection to hospitalisation, the time from hospitalisation to death, and the time from infection to death.

### Data preparation

The two datasets used in this study were merged and split in order to measure the three quantities of interest. Subsequently, rows with missing values and duplicates were dropped. As the datasets were anonymised, it was assumed that if two lines had the same local authority area, sex, age, start date and end date, then they referred to the same person. Additionally, the data was filtered to remove erroneous negative time-delay periods and extreme outliers prior to model fitting. The data were then split into distinct epidemiological periods: the first wave (January to May), the summer (June to August), the second wave (September to November), and the third wave (December to January). The periods were defined by clear distributional changes in the time delays that had an evident seasonality with distinct peaks in prevalence and hospital admissions:

- 1^st^ Period: The first period was characterised by a sharp increase of SARS-CoV-2 incidence that peaked at 280000 [10], which across the period led to daily hospital admissions having a median of 1466 [1] and this precipitated the first national lockdown.
- 2^nd^ Period: The second period saw a loosening of NPIs with the median for daily hospital admissions dropping to 162 [1] and incidence estimates peaking at 10700 [10].
- 3^rd^ Period: The third period was characterised by the introduction of tiers that determined the extent of the NPIs that were required locally. It saw an increase in the median for daily hospital admissions to 1025 [1] and a peak incidence estimate of 66800 [10].
- 4^th^ Period: The middle of the fourth period saw the start of a national lockdown with the highest median for daily hospital admissions of 2529 and incidence estimates peaking at 157000 [10].

In addition, in order to assess the dependence of time delay on gender and age, we split the combined data by ten-year age bands and gender using the data from January 2020 to November 2020. These dates were selected so that the full distribution of hospitalisations and deaths had been observed. We did not have reliable data on infection to symptom onset so this was informed by a literature estimate [5]. We then calculated for these periods two categories based on the date that the data was collected.

Category A:
  (a) infection to hospitalisation by date of symptom onset
  (b) hospitalisation to death by date of hospitalisation
  (c) infection to death by date of symptom onset
Category B:
  (a) infection to hospitalisation by date of hospitalisation
  (b) hospitalisation to death by date of death
  (c) infection to death by date of death

For each time interval we then fit the Lognormal, Gamma and Weibull distributions to a doubly interval censored likelihood function that was run through Monte Carlo sampling. This was used to address the inherently ‘coarse’ [8] nature of this data, in part due to how it was recorded.

### Time delay distribution modelling




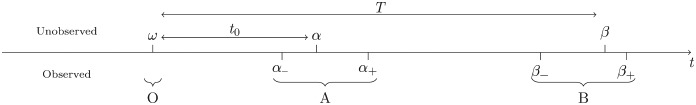




We define two events, A and B, and the times at which these events occur, *α* and *β*, with *α* < *β*. However, *α* and *β* are not known precisely: *α* ∈ [*α*_−_, *α*_+_], *β* ∈ [*β*_−_, *β*_+_]. In addition, let O be an unobserved event that occurs a time *t*_0_ prior to A. The probability density function governing the time from O to A is *p*(*t*_0_). Let the time between events O and B be *T*, a continuous random variable with probability density function *f*(*t*;***θ***) dependent on parameters ***θ***. We express the joint probability of all three events as *p*(O, A, B) = *p*(B|O, A) ⋅ *p*(O|A) ⋅ *p*(A) = *f*(*β* − *α* + *t*_0_;***θ***) ⋅ *p*(*t*_0_) ⋅ *p*(*α*). In the absence of information informing *p*(*α*), let it be a uniform distribution. In other words,

$$\begin{array}{cc} p(\alpha ) & =\{\begin{array}{cc} \frac{1}{\alpha_{+}-\alpha_{-}} & \alpha_{-}\leq \alpha \leq \alpha_{+}\\ 0 & \text{otherwise} \end{array} \end{array}$$

Then, we can express the likelihood of ***θ*** and an observed data point *X*^*i*^,$\left(\alpha_{-}^{i},\alpha_{+}^{i},\beta_{-}^{i},\beta_{+}^{i}\right)$, as

$$\begin{array}{c} L(\theta ,X^{i})=\int_{\alpha_{-}^{i}}^{\alpha_{+}^{i}}\int_{\beta_{-}^{i}}^{\beta_{+}^{i}}\int_{0}^{\infty }f(\beta^{i}-\alpha^{i}+t_{0};\theta )p(t_{0})\;dt_{0}\;d\beta^{i}\;d\alpha^{i}. \end{array}$$ (1)

For multiple data points ***X*** = {*X*^*i*^}, the likelihood is

$$\begin{array}{c} L(\theta ,X)=\prod_{i}L(\theta ,X^{i}) \end{array}$$ (2)

and a Hamiltonian Markov chain Monte Carlo method is used within Stan [11] to find the distributions of ***θ***, given the observed data.

Within the context of this paper, the events O, A and B refer to the quantities in Table 1. We use the literature [5] to inform the time between O and A as *p*(*t*_0_) ∼ Lognormal (1.63, 0.50). In the specific case that the time we want to measure is in fact A to B rather than O to B, we can let *p*(*t*_0_) = *δ*(*t*_0_) where *δ* here refers to a half delta function defined on $t_{0}\in R_{0}^{+}$.

**Table 1:** Events O, A and B for the three time delay quantities.

	O	A	B
Infection to hospitalisation	Infection	Symptom onset	Hospitalisation
Hospitalisation to death	N/A	Hospitalisation	Death
Infection to death	Infection	Symptom onset	Death

In order to account for the right truncation present within the most recent portion of the dataset, we use a modified probability density function *f*_*RT*_ that accounts for this [6]

$$\begin{array}{c} f_{RT}(\beta -\alpha +t_{0};\theta )=\frac{f(\beta -\alpha +t_{0};\theta )}{\int_{0}^{\text{max}(\beta_{+}^{i})-\alpha }\frac{r\text{exp}(-ru)}{1-\text{exp}(-ru)}F(\text{max}(\beta_{+}^{i})-\alpha -u;\theta )\;du} \end{array}$$ (3)

where $F(t;\theta )=\int_{0}^{t}f(\tau ;\theta )\;d\tau$ is the cumulative probability function of *f* and *r* is the exponential growth rate of type A events. In this paper there are two categories of type A event: symptom onset and admission to hospital. In order to calculate the growth rate, a negative binomial was fitted to modelled incidence [12] for symptom onset, and publicly available admissions data [1] for hospitalisations.

### Models and assessing performance

For each set of data points, the probability density function *f* is taken to follow the Lognormal, Gamma and Weibull distributions as these are commonly used for survival data. Their probability density functions, defined for *x* ≥ 0, are as follows

$$\begin{array}{c} \text{Lognormal}:f(x;\alpha ,\beta )=\frac{1}{x\sqrt{2\pi \beta^{2}}}\;\text{exp}(-\frac{{\left(\text{ln}\;x-\alpha \right)}^{2}}{2\beta^{2}})\\ \text{Gamma}:f(x;\alpha ,\beta )=\frac{1}{\Gamma (\alpha )\beta^{\alpha }}\;x^{\alpha -1}\;\text{exp}(-\frac{x}{\beta })\\ \text{Weibull}:f(x;\alpha ,\beta )=\alpha \beta^{-\alpha }x^{\alpha -1}\;\text{exp}(-{\left(\frac{x}{\beta }\right)}^{\alpha }). \end{array}$$

We calculated the leave-one-out cross-validation (LOO) using Pareto-smoothed importance sampling (PSIS) and the widely applicable information criterion (WAIC) [13] scores for each model to compare the accuracy of the fitted Bayesian models. The WAIC score is asymptotically equivalent to LOO and can be thought of as an approximation [14]. Therefore, LOO scores were used in conjunction with Pareto *k* diagnostics and the R-hat convergence diagnostics to assess the best model fit. Most desirable is the lowest LOO score alongside a Pareto score where *k* ≤ 0.7 and an $\hat{R}\leq 1.05$ [13].

## Results

We present two sets of results in this paper: (i) the evolution of the times to clinical outcome over the course of the pandemic, and (ii) the variation in those times by sex and age group. The times to clinical outcome that are measured are infection to hospitalisation, hospitalisation to death, and infection to death. The modelled estimates that were of primary interest were informed by category A (see section on data preparation) rather than by category B because these estimates are not influenced by historical infections in the defined periods. We report category B results (Tables A1-A3 in the S1 Appendix) as they may have utility for epidemiological modelling and when assessing external factors, such as the impact of healthcare pressure, because it captures those individuals that died or were hospitalised in that period. The choice of which date category to use impacts the whole time delay distribution. The right tail of the distribution using category B data may capture some individuals infected in an earlier period whereas, using category A to inform estimates may capture some hospitalisations and deaths from a later period. We were not aware of any selection bias for individuals that were included in the datasets used for modelling although, ascertainment bias for cases would be more evident in the earlier periods when testing capacity was more limited. The sample of individuals that have symptom onset included in the death data line list pertains to reporting practices of certain testing laboratories. In the SARI dataset highly detailed data is collected for a subset of NHS Trusts, which includes symptom onset. Tables 2 and 4 show that the Lognormal is a better fit for the infection to hospitalisation and infection to death distributions whereas, Table 3 illustrates that Weibull is a better fit for the hospitalisation to death distribution.

**Table 2:** Results for the time from infection to hospitalisation, segmented by symptom onset date.

Period	N	Model	Mean	SD	*α*	*β*	LOOIC	%(*k*≤0.7)
01Jan2020 to 31May2020	4328	Lognormal	8.01 (7.89, 8.14)	4.16 (4.03, 4.30)	1.96 (1.95, 1.98)	0.49 (0.48, 0.50)	24022	98
		Gamma	8.13 (8.02, 8.25)	3.97 (3.88, 4.07)	4.19 (4.01, 4.38)	0.52 (0.49, 0.54)	24309	98
		Weibull	8.21 (8.08, 8.33)	4.26 (4.17, 4.34)	2.02 (1.97, 2.06)	9.26 (9.12, 9.41)	24726	96
01Jun2020 to 31Aug2020	135	Lognormal	9.21 (8.34, 10.15)	6.22 (5.18, 7.45)	2.03 (1.93, 2.13)	0.61 (0.55, 0.68)	826	99
		Gamma	10.07 (9.07, 11.17)	7.04 (6.14, 8.07)	2.07 (1.68, 2.51)	0.21 (0.16, 0.25)	875	100
		Weibull	10.20 (8.99, 11.48)	8.44 (7.36, 9.72)	1.22 (1.10, 1.34)	10.87 (9.51, 12.30)	896	99
01Sep2020 to 30Nov2020	1052	Lognormal	9.70 (9.44, 9.97)	4.44 (4.16, 4.73)	2.18 (2.15, 2.20)	0.44 (0.41, 0.46)	6096	95
		Gamma	9.75 (9.49, 10.01)	4.29 (4.08, 4.52)	5.17 (4.70, 5.67)	0.53 (0.48, 0.58)	6134	98
		Weibull	9.78 (9.51, 10.05)	4.55 (4.37, 4.74)	2.28 (2.17, 2.39)	11.04 (10.74, 11.34)	6214	94
01Dec2020 to 20Jan2021	489	Lognormal	8.91 (8.59, 9.27)	3.90 (3.56, 4.28)	2.10 (2.06, 2.14)	0.42 (0.39, 0.45)	2742	93
		Gamma	8.97 (8.63, 9.32)	3.75 (3.47, 4.05)	5.75 (4.93, 6.64)	0.64 (0.55, 0.74)	2748	98
		Weibull	9.02 (8.66, 9.39)	3.86 (3.63, 4.11)	2.51 (2.32, 2.70)	10.17 (9.77, 10.56)	2773	89
		Lognormal*	9.72 (9.31, 10.15)	4.04 (3.57, 4.57)	2.19 (2.15, 2.24)	0.40 (0.36, 0.44)	4847	88
		Gamma*	†	†	†	†	†	†
		Weibull*	9.75 (9.36, 10.14)	3.93 (3.66, 4.22)	2.68 (2.46, 2.91)	10.96 (10.54, 11.41)	4890	91

For the last time period from 1 December 2020 to 20 January 2021, the right truncated model was run as well. 90% credible intervals are quoted

*Model run with right truncation using *r* = 0.0173.

^†^Model did not converge $\left(\hat{R}>1.05\right)$.

**Table 3:** Results for the time from hospitalisation to death, segmented by hospitalisation date.

Period	N	Model	Mean	SD	*α*	*β*	LOOIC	%(*k*≤0.7)
01Jan2020 to 31May2020	28611	Lognormal	11.12 (10.98, 11.25)	14.63 (14.31, 14.94)	1.91 (1.90, 1.92)	1.00 (0.99, 1.01)	192080	96
		Gamma	10.29 (10.20, 10.38)	9.51 (9.41, 9.61)	1.17 (1.15, 1.19)	0.11 (0.11, 0.12)	190324	100
		Weibull	10.28 (10.19, 10.38)	9.36 (9.25, 9.47)	1.10 (1.09, 1.11)	10.66 (10.56, 10.76)	190288	100
01Jun2020 to 31Aug2020	1700	Lognormal	15.89 (15.02, 16.80)	23.46 (21.30, 25.91)	2.19 (2.14, 2.23)	1.07 (1.04, 1.11)	12609	97
		Gamma	14.62 (14.06, 15.20)	14.31 (13.64, 15.02)	1.04 (0.99, 1.10)	0.07 (0.07, 0.08)	12527	100
		Weibull	14.63 (14.08, 15.21)	14.43 (13.76, 15.14)	1.01 (0.98, 1.05)	14.72 (14.10, 15.33)	12528	100
01Sep2020 to 30Nov2020	16641	Lognormal	16.43 (16.19, 16.67)	19.52 (19.02, 20.04)	2.36 (2.35, 2.37)	0.94 (0.93, 0.95)	124441	97
		Gamma	14.99 (14.84, 15.15)	12.35 (12.18, 12.52)	1.47 (1.45, 1.50)	0.10 (0.10, 0.10)	122198	100
		Weibull	14.96 (14.81, 15.11)	11.62 (11.46, 11.78)	1.30 (1.28, 1.31)	16.19 (16.02, 16.36)	121915	100
01Dec2020 to 20Jan2021	16903	Lognormal	10.08 (9.94, 10.21)	11.30 (11.03, 11.58)	1.90 (1.89, 1.91)	0.90 (0.89, 0.91)	110011	95
		Gamma	9.33 (9.24, 9.43)	7.64 (7.54, 7.75)	1.49 (1.46, 1.52)	0.16 (0.16, 0.16)	108147	100
		Weibull	9.31 (9.21, 9.40)	7.17 (7.08, 7.27)	1.31 (1.29, 1.32)	10.09 (9.98, 10.19)	107815	100
	1000^⋆^	Lognormal*	26.44 (20.80, 34.06)	51.50 (35.00, 75.27)	2.49 (2.36, 2.65)	1.24 (1.15, 1.34)	10656	96
		Gamma*	†	†	†	†	†	†
		Weibull*	12.41 (11.49, 13.54)	10.20 (9.11, 11.55)	1.23 (1.16, 1.30)	13.26 (12.32, 14.37)	10605	100

For the last time period from 1 December 2020 to 20 January 2021, the right truncated model was run as well. 90% credible intervals are quoted.

*Model run with right truncation using *r* = 0.0278.

^⋆^Due to computational constraints, we used a randomly selected subsample of 1000 people for the right truncated model.

^†^Model did not converge $\left(\hat{R}>1.05\right)$.

**Table 4:** Results for the time from infection to death, segmented by symptom onset date.

Period	N	Model	Mean	SD	*α*	*β*	LOOIC	%(*k*≤0.7)
01Jan2020 to 31May2020	5378	Lognormal	19.61 (19.34, 19.89)	11.82 (11.51, 12.16)	2.82 (2.81, 2.83)	0.56 (0.55, 0.57)	39680	100
		Gamma	19.88 (19.62, 20.15)	11.35 (11.11, 11.60)	3.07 (2.96, 3.17)	0.15 (0.15, 0.16)	40340	100
		Weibull	20.02 (19.73, 20.32)	12.61 (12.39, 12.84)	1.63 (1.60, 1.65)	22.36 (22.03, 22.71)	41071	100
01Jun2020 to 31Aug2020	170	Lognormal	24.69 (22.58, 27.05)	17.42 (14.82, 20.68)	3.00 (2.91, 3.09)	0.63 (0.58, 0.70)	1368	100
		Gamma	25.29 (23.33, 27.45)	16.59 (14.70, 18.63)	2.35 (1.95, 2.77)	0.09 (0.08, 0.11)	1393	100
		Weibull	25.56 (23.32, 27.99)	18.14 (16.32, 20.19)	1.43 (1.30, 1.57)	28.12 (25.52, 30.91)	1411	100
01Sep2020 to 30Nov2020	1335	Lognormal	23.00 (22.45, 23.55)	12.04 (11.42, 12.68)	3.01 (2.99, 3.04)	0.49 (0.47, 0.51)	10081	100
		Gamma	22.95 (22.45, 23.47)	11.22 (10.79, 11.67)	4.19 (3.91, 4.47)	0.18 (0.17, 0.20)	10103	100
		Weibull	23.02 (22.50, 23.56)	11.56 (11.20, 11.94)	2.09 (2.02, 2.16)	25.99 (25.40, 26.60)	10193	99
01Dec2020 to 20Jan2021	650	Lognormal	17.40 (16.94, 17.88)	6.81 (6.35, 7.29)	2.79 (2.76, 2.81)	0.38 (0.36, 0.40)	4330	98
		Gamma	17.39 (16.94, 17.85)	6.58 (6.19, 7.01)	7.01 (6.23, 7.81)	0.40 (0.36, 0.45)	4333	100
		Weibull	17.43 (16.96, 17.89)	6.72 (6.41, 7.05)	2.81 (2.65, 2.97)	19.57 (19.06, 20.07)	4362	93
		Lognormal*	22.68 (21.97, 23.35)	6.05 (5.82, 6.25)	3.09 (3.06, 3.12)	0.26 (0.26, 0.27)	6516	86
		Gamma*	†	†	†	†	†	†
		Weibull*	21.81 (21.11, 22.54)	6.85 (6.49, 7.28)	3.53 (3.33, 3.69)	24.23 (23.45, 25.04)	6766	94

For the last time period from 1 December 2020 to 20 January 2021, the right truncated model was run as well. 90% credible intervals are quoted.

*Model run with right truncation using *r* = 0.0173.

^†^Model did not converge $\left(\hat{R}>1.05\right)$.

### Variation in time over the pandemic

Tables 2–4 show the distributions of these times for the four distinct periods described in the Methods section. There is a consistent age structure for the hospitalisations and deaths, which is highly skewed towards the older demographics, irrespective of the temporal period. Noteworthy is the result that the mean time from infection to hospitalisation has remained the most constant of the three time delay quantities. This contrasts with noticeable increases observed in the time from hospitalisation to death and infection to death over the summer and early autumn months of 2020 when prevalence was lower, with declines observed in the most recent period.

### Variation in time by sex and age

**Fig 1:**
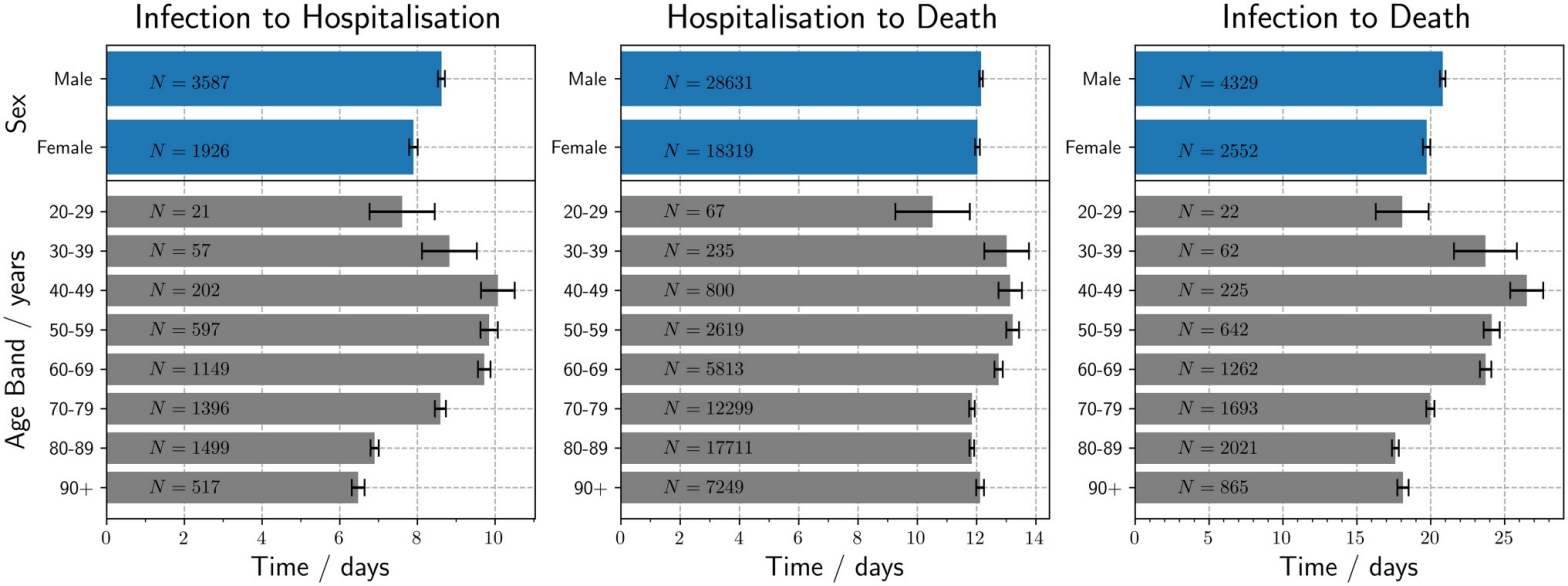
Bar charts of the times to clinical outcome by age and sex, with standard errors. The data were filtered for dates between January 2020 to November 2020 and *N* on each bar represents the number of patients within each group.

Additionally, modelled results by sex and age can be seen in Tables 5–7. Fig 1 illustrates that men had a longer time delay distribution than women for infection to hospitalisation; however, there was no statistically significant difference in the time from hospitalisation to death between the sexes. For the variation by age, the mean time from infection to hospitalisation and death increases from those in their twenties to peak in patients in their forties, followed by a steady reduction with increasing patient age until 80–89. The variation observed within the time from hospitalisation to death was more modest; nevertheless, middle-aged patients displayed the longest times as observed in infection to death. Results for people under the age of 20 were discarded because there were too few patients for a meaningful measurement of their epidemiological characteristics. Males have a greater time from infection to hospitalisation, which was statistically significant, with a p-value of 5.0 × 10^−15^ using a Mann-Whitney-Wilcoxon. This same distinction between males and females is not found for the time delay in hospitalisation to death with a p-value of 0.93.

**Table 5:** Results for the time from infection to hospitalisation by sex and age.

Sex/Age	N	Model	Mean	SD	*α*	*β*	LOOIC	%(*k*≤0.7)
Male	3587	Lognormal	8.62 (8.48, 8.77)	4.49 (4.33, 4.66)	2.03 (2.02, 2.05)	0.49 (0.48, 0.50)	20429	98
		Gamma	8.73 (8.59, 8.87)	4.31 (4.19, 4.43)	4.11 (3.91, 4.31)	0.47 (0.45, 0.49)	20660	99
		Weibull	8.75 (8.59, 8.89)	4.81 (4.71, 4.92)	1.89 (1.85, 1.93)	9.85 (9.68, 10.02)	21115	98
Female	1926	Lognormal	7.90 (7.72, 8.08)	4.01 (3.83, 4.21)	1.95 (1.93, 1.98)	0.48 (0.46, 0.50)	10602	97
		Gamma	8.04 (7.87, 8.21)	3.91 (3.77, 4.06)	4.24 (3.96, 4.53)	0.53 (0.49, 0.56)	10759	98
		Weibull	8.08 (7.89, 8.27)	4.37 (4.25, 4.50)	1.93 (1.87, 1.98)	9.11 (8.90, 9.33)	11018	94
20–29	21	Lognormal	7.61 (6.26, 9.00)	2.81 (1.77, 4.28)	1.96 (1.74, 2.15)	0.36 (0.22, 0.52)	111	52
		Gamma	7.97 (6.58, 9.52)	3.01 (1.91, 4.51)	8.53 (3.27, 18.11)	1.07 (0.41, 2.20)	111	81
		Weibull	8.13 (6.70, 9.56)	2.74 (1.80, 3.89)	3.52 (2.07, 5.55)	9.03 (7.54, 10.52)	112	81
30–39	57	Lognormal	8.83 (7.71, 10.02)	4.79 (3.69, 6.19)	2.04 (1.90, 2.18)	0.51 (0.41, 0.61)	335	98
		Gamma	9.12 (7.97, 10.34)	4.70 (3.76, 5.85)	3.92 (2.56, 5.63)	0.43 (0.28, 0.62)	337	96
		Weibull	9.24 (8.09, 10.45)	4.80 (4.01, 5.82)	2.04 (1.65, 2.48)	10.41 (9.09, 11.79)	341	98
40–49	202	Lognormal	10.07 (9.36, 10.80)	5.58 (4.81, 6.48)	2.17 (2.10, 2.25)	0.52 (0.46, 0.57)	1228	99
		Gamma	10.17 (9.51, 10.86)	5.21 (4.65, 5.84)	3.85 (3.11, 4.70)	0.38 (0.31, 0.46)	1234	100
		Weibull	10.23 (9.53, 10.95)	5.28 (4.80, 5.84)	2.04 (1.82, 2.26)	11.54 (10.75, 12.36)	1244	99
50–59	597	Lognormal	9.85 (9.48, 10.22)	4.77 (4.37, 5.21)	2.18 (2.14, 2.22)	0.46 (0.43, 0.49)	3522	97
		Gamma	9.87 (9.52, 10.23)	4.50 (4.20, 4.81)	4.83 (4.24, 5.47)	0.49 (0.43, 0.56)	3527	99
		Weibull	9.92 (9.56, 10.72)	4.57 (4.32, 4.83)	2.31 (2.16, 2.46)	11.19 (10.80, 11.60)	3552	95
60–69	1149	Lognormal	9.72 (9.47, 9.99)	4.77 (4.49, 5.08)	2.17 (2.14, 2.19)	0.46 (0.44, 0.49)	6755	97
		Gamma	9.77 (9.50, 10.03)	4.59 (4.37, 4.81)	4.55 (4.15, 4.97)	0.47 (0.42, 0.51)	6799	99
		Weibull	9.75 (9.49, 10.03)	5.05 (4.87, 5.23)	2.02 (1.95, 2.10)	11.01 (10.70, 11.33)	6926	97
70–79	1396	Lognormal	8.59 (8.36, 8.83)	4.52 (4.27, 4.79)	2.03 (2.00, 2.06)	0.49 (0.47, 0.52)	7956	98
		Gamma	8.72 (8.51, 8.94)	4.39 (4.21, 4.58)	3.95 (3.66, 4.26)	0.45 (0.42, 0.49)	8073	99
		Weibull	8.74 (8.48, 8.99)	5.03 (4.86, 5.20)	1.80 (1.74, 1.86)	9.82 (9.53, 10.11)	8287	98
80–89	1499	Lognormal	6.90 (6.72, 7.07)	3.19 (3.03, 3.36)	1.83 (1.81, 1.86)	0.44 (0.42, 0.46)	7729	94
		Gamma	7.06 (6.89, 7.23)	3.17 (3.04, 3.31)	4.96 (4.57, 5.34)	0.70 (0.65, 0.76)	7866	93
		Weibull	7.14 (6.95, 7.33)	3.59 (3.47, 3.72)	2.09 (2.01, 2.17)	8.06 (7.84, 8.28)	8081	86
90+	517	Lognormal	6.48 (6.21, 6.75)	2.98 (2.74, 3.24)	1.77 (1.73, 1.82)	0.44 (0.41, 0.47)	2605	93
		Gamma	6.70 (6.42, 6.98)	3.13 (2.92, 3.36)	4.61 (4.05, 5.19)	0.69 (0.61, 0.78)	2692	93
		Weibull	6.75 (6.43, 7.09)	4.00 (3.79, 4.22)	1.74 (1.65, 1.83)	7.58 (7.21, 7.97)	2837	95

The data were filtered for symptom onset dates between January 2020 to November 2020. 90% credible intervals are quoted.

**Table 6:** Results for the time from hospitalisation to death by sex and age.

Sex/Age	N	Model	Mean	SD	*α*	*β*	LOOIC	%(*k*≤0.7)
Male	28631	Lognormal	13.22 (13.07, 13.38)	17.07 (16.70, 17.44)	2.09 (2.08, 2.10)	0.99 (0.98, 1.00)	201970	97
		Gamma	12.16 (12.05, 12.26)	10.88 (10.77, 11.00)	1.25 (1.23, 1.26)	0.10 (0.10, 0.10)	199641	100
		Weibull	12.15 (12.05, 12.25)	10.60 (10.48, 10.72)	1.15 (1.14, 1.16)	12.76 (12.65, 12.87)	199552	100
Female	18319	Lognormal	13.26 (13.05, 13.47)	18.26 (17.76, 18.79)	2.05 (2.04, 2.07)	1.03 (1.02, 1.04)	129323	96
		Gamma	12.04 (11.91, 12.17)	11.20 (11.05, 11.36)	1.16 (1.14, 1.18)	0.10 (0.09, 0.10)	127640	100
		Weibull	12.03 (11.90, 12.16)	10.90 (10.74, 11.06)	1.11 (1.09, 1.12)	12.48 (12.34, 12.63)	127576	100
20–29	67	Lognormal	10.44 (8.31, 13.15)	14.43 (9.66, 21.34)	1.81 (1.59, 2.02)	1.02 (0.88, 1.18)	462	93
		Gamma	10.39 (8.52, 12.61)	10.44 (8.13, 13.51)	1.02 (0.72, 1.34)	0.10 (0.07, 0.14)	450	100
		Weibull	10.51 (8.65, 12.71)	10.02 (7.74, 13.22)	1.07 (0.88, 1.26)	10.69 (8.62, 12.96)	450	100
30–39	235	Lognormal	13.56 (12.04, 15.33)	16.97 (13.74, 21.04)	2.14 (2.03, 2.24)	0.97 (0.89, 1.05)	1685	97
		Gamma	12.99 (11.76, 14.28)	11.54 (10.25, 13.00)	1.27 (1.09, 1.47)	0.10 (0.08, 0.12)	1671	100
		Weibull	13.01 (11.86, 14.30)	11.33 (10.06, 12.82)	1.15 (1.05, 1.26)	13.67 (12.37, 15.08)	1671	100
40–49	800	Lognormal	14.56 (13.52, 15.69)	19.80 (17.34, 22.59)	2.16 (2.09, 2.22)	1.02 (0.98, 1.07)	5821	96
		Gamma	13.13 (12.44, 13.85)	11.98 (11.21, 12.80)	1.20 (1.11, 1.30)	0.09 (0.08, 0.10)	5712	100
		Weibull	13.13 (12.49, 13.79)	11.47 (10.75, 12.24)	1.15 (1.09, 1.21)	13.79 (13.07, 14.51)	5705	100
50–59	2619	Lognormal	14.47 (13.91, 15.04)	18.26 (17.04, 19.56)	2.20 (2.16, 2.23)	0.98 (0.95, 1.00)	18962	96
		Gamma	13.24 (12.88, 13.61)	11.54 (11.14, 11.95)	1.32 (1.26, 1.38)	0.10 (0.09, 0.10)	18677	100
		Weibull	13.22 (12.87, 13.58)	11.07 (10.68, 11.47)	1.20 (1.17, 1.23)	14.05 (13.65, 14.44)	18656	100
60–69	5813	Lognormal	13.93 (13.58, 14.28)	17.51 (16.75, 18.31)	2.16 (2.14, 2.18)	0.97 (0.96, 0.99)	41618	96
		Gamma	12.75 (12.52, 13.00)	11.12 (10.86, 11.39)	1.32 (1.28, 1.36)	0.10 (0.10, 0.11)	41019	100
		Weibull	12.74 (12.51, 12.98)	10.70 (10.45, 10.96)	1.20 (1.17, 1.22)	13.53 (13.27, 13.80)	40977	100
70–79	12299	Lognormal	12.85 (12.63, 13.09)	16.55 (16.03, 17.11)	2.06 (2.05, 2.08)	0.99 (0.98, 1.00)	86074	96
		Gamma	11.85 (11.69, 12.01)	10.62 (10.44, 10.81)	1.24 (1.22, 1.27)	0.11 (0.10, 0.11)	85139	100
		Weibull	11.84 (11.69, 12.00)	10.37 (10.19, 10.55)	1.14 (1.13, 1.16)	12.43 (12.27, 12.60)	85109	100
80–89	17711	Lognormal	12.92 (12.72, 13.12)	17.48 (17.01, 17.96)	2.04 (2.03, 2.05)	1.02 (1.01, 1.03)	124108	96
		Gamma	11.84 (11.71, 11.98)	10.98 (10.83, 11.14)	1.16 (1.14, 1.18)	0.10 (0.10, 0.10)	122803	100
		Weibull	11.84 (11.71, 11.97)	10.77 (10.62, 10.92)	1.10 (1.09, 1.11)	12.27 (12.13, 12.42)	122771	100
90+	7249	Lognormal	13.39 (13.06, 13.73)	18.38 (17.58, 19.23)	2.06 (2.04, 2.08)	1.03 (1.01, 1.05)	51343	96
		Gamma	12.13 (11.91, 12.35)	11.22 (10.98, 11.49)	1.17 (1.14, 1.20)	0.10 (0.09, 0.10)	50604	100
		Weibull	12.12 (11.90, 12.33)	10.88 (10.63, 11.14)	1.12 (1.10, 1.13)	12.61 (12.37, 12.84)	50571	100

The data were filtered for hospitalisation dates between January 2020 to November 2020. 90% credible intervals are quoted.

**Table 7:** Results for the time from infection to death by sex and age.

Sex/Age	N	Model	Mean	SD	*α*	*β*	LOOIC	%(*k*≤0.7)
Male	4329	Lognormal	20.81 (20.51, 21.12)	12.09 (11.74, 12.48)	2.89 (2.88, 2.90)	0.54 (0.53, 0.55)	32285	100
		Gamma	20.95 (20.66, 21.24)	11.46 (11.20, 11.72)	3.35 (3.22, 3.47)	0.16 (0.15, 0.17)	32662	100
		Weibull	21.06 (20.74, 21.39)	12.53 (12.29, 12.78)	1.73 (1.70, 1.76)	23.63 (23.26, 24.01)	33200	99
Female	2552	Lognormal	19.72 (19.31, 20.14)	12.26 (11.76, 12.79)	2.82 (2.80, 2.84)	0.57 (0.56, 0.59)	18941	100
		Gamma	20.01 (19.62, 20.41)	11.72 (11.37, 12.08)	2.92 (2.78, 3.06)	0.15 (0.14, 0.15)	19264	100
		Weibull	20.16 (19.72, 20.60)	12.95 (12.64, 13.27)	1.59 (1.56, 1.63)	22.48 (21.96, 22.99)	19595	100
20–29	22	Lognormal	18.07 (15.23, 21.03)	7.67 (5.32, 11.07)	2.80 (2.63, 2.96)	0.40 (0.30, 0.54)	156	100
		Gamma	18.43 (15.57, 21.38)	7.76 (5.63, 10.57)	6.18 (3.25, 10.43)	0.34 (0.17, 0.56)	155	100
		Weibull	18.60 (15.71, 21.48)	7.70 (5.96, 10.09)	2.67, (1.89, 3.53)	20.89 (17.70, 24.03)	156	100
30–39	62	Lognormal	23.70 (20.48, 27.48)	16.74 (12.89, 21.92)	2.96 (2.81, 3.10)	0.63 (0.54, 0.74)	501	100
		Gamma	24.45 (21.35, 27.85)	16.12 (13.36, 19.44)	2.35 (1.73, 3.10)	0.10 (0.07, 0.13)	509	100
		Weibull	24.79 (21.46, 28.45)	17.42 (14.74, 20.80)	1.45 (1.25, 1.68)	27.29 (23.44, 31.54)	515	100
40–49	225	Lognormal	26.50 (24.72, 28.41)	16.63 (14.54, 19.00)	3.11 (3.04, 3.18)	0.58 (0.53, 0.63)	1812	100
		Gamma	26.47 (24.81, 28.15)	15.07 (13.65, 16.62)	3.11 (2.64, 3.63)	0.12 (0.10, 0.14)	1820	100
		Weibull	26.64 (24.89, 28.42)	15.46 (14.24, 16.87)	1.79 (1.63, 1.94)	29.93 (27.91, 31.96)	1834	100
50–59	642	Lognormal	24.13 (23.28, 25.05)	13.25 (12.28, 14.32)	3.05 (3.02, 3.09)	0.51 (0.49, 0.54)	4946	100
		Gamma	24.12 (23.33, 24.96)	12.37 (11.68, 13.09)	3.81 (3.45, 4.19)	0.16 (0.14, 0.17)	4968	100
		Weibull	24.21 (23.36, 25.10)	13.01 (12.42, 13.67)	1.94 (1.84, 2.04)	27.30 (26.32, 28.30)	5025	99
60–69	1262	Lognormal	23.71 (23.10, 24.35)	13.35 (12.63, 14.13)	3.03 (3.00, 3.05)	0.52 (0.51, 0.54)	9707	100
		Gamma	23.73 (23.13, 24.35)	12.49 (11.97, 13.03)	3.62 (3.37, 3.87)	0.15 (0.14, 0.16)	9768	100
		Weibull	23.82 (23.18, 24.46)	13.36 (12.91, 13.85)	1.85 (1.79, 1.91)	26.82 (26.07, 27.55)	9898	100
70–79	1693	Lognormal	19.97 (19.52, 20.44)	10.92 (10.40, 11.45)	2.86 (2.84, 2.89)	0.51 (0.49, 0.53)	12385	100
		Gamma	20.13 (19.70, 20.57)	10.53 (10.16, 10.92)	3.66 (3.43, 3.88)	0.18 (0.17, 0.19)	12537	100
		Weibull	20.21 (19.74, 20.70)	11.72 (11.37, 12.08)	1.78 (1.73, 1.84)	22.72 (22.18, 23.27)	12780	99
80–89	2021	Lognormal	17.61 (17.23, 17.99)	10.29 (9.84, 10.78)	2.72 (2.70, 2.74)	0.54 (0.53, 0.56)	14418	100
		Gamma	17.95 (17.57, 18.34)	10.16 (9.82, 10.52)	3.12 (2.96, 3.29)	0.17 (0.16, 0.18)	14733	100
		Weibull	18.10 (17.66, 18.54)	11.58 (11.24, 11.92)	1.60 (1.56, 1.64)	20.18 (19.68, 20.69)	15056	100
90+	865	Lognormal	18.12 (17.50, 18.77)	11.50 (10.74, 12.32)	2.73 (2.69, 2.76)	0.58 (0.56, 0.61)	6296	100
		Gamma	18.65 (18.01, 19.30)	11.44 (10.84, 12.05)	2.66 (2.46, 2.88)	0.14 (0.13, 0.16)	6466	100
		Weibull	18.85 (18.13, 19.59)	13.03 (12.46, 13.67)	1.47 (1.41, 1.53)	20.83 (20.00, 21.66)	6596	100

The data were filtered for symptom onset dates between January 2020 to November 2020. 90% credible intervals are quoted.

## Discussion

The impact of SARS-CoV-2 between subgroups of the population and across periods defined by distinct temporal epidemiological trends is significant in furthering understanding of the virus and how we might expect it to change over time. Understanding the clinical time delays and the impetus that drive the changes in these distributions will help to untangle extrinsic pressure from any further phenotypic changes we encounter in the virus. This will help to inform more impactful policy decisions on the containment and the suppression of transmission and allow for a clearer understanding of variants of concern.

**Fig 2:**
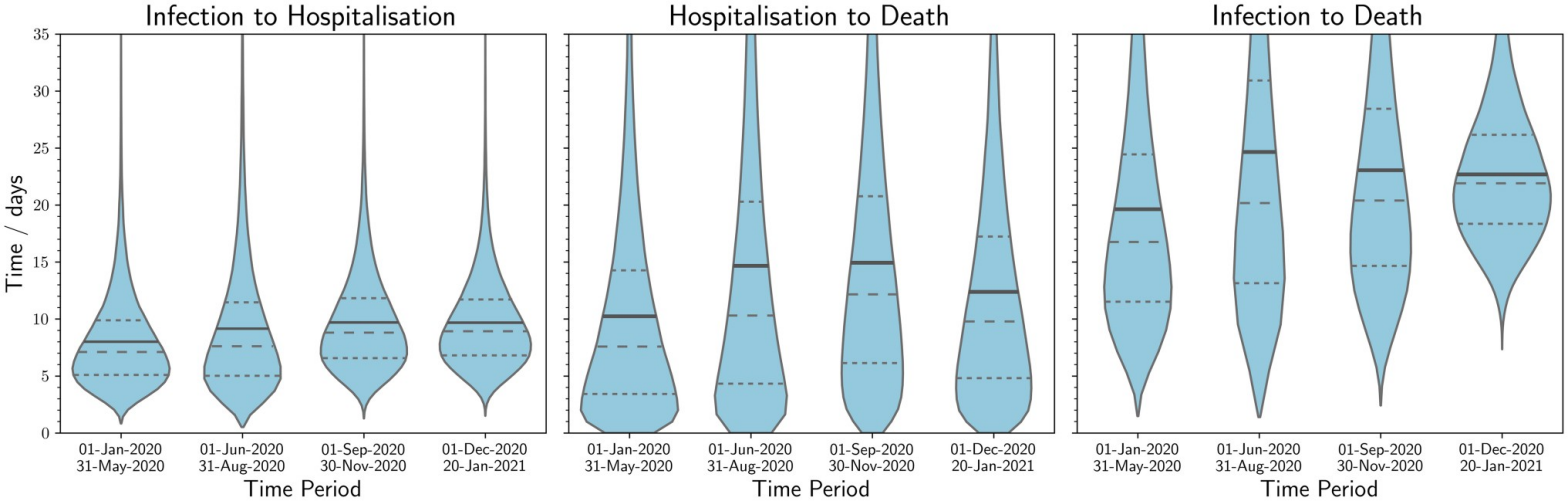
Violin plots of the best fit modelled distributions of times to clinical outcome over the course of the pandemic for category A. The quartiles for each distribution are shown as dashed lines and the solid line corresponds to the mean. In this chart, the data are segmented in time by the former of the two events: symptom onset date for both infection to hospitalisation and infection to death, and hospitalisation date for hospitalisation to death. For the last time period (1 December 2020 to 20 January 2021), the right truncation equation was used. The mean time from infection to hospitalisation has remained relatively stable. For both hospitalisation to death and infection to death, the mean time was lowest in the first wave, and there was a marked increase over the summer months.

**Fig 3:**
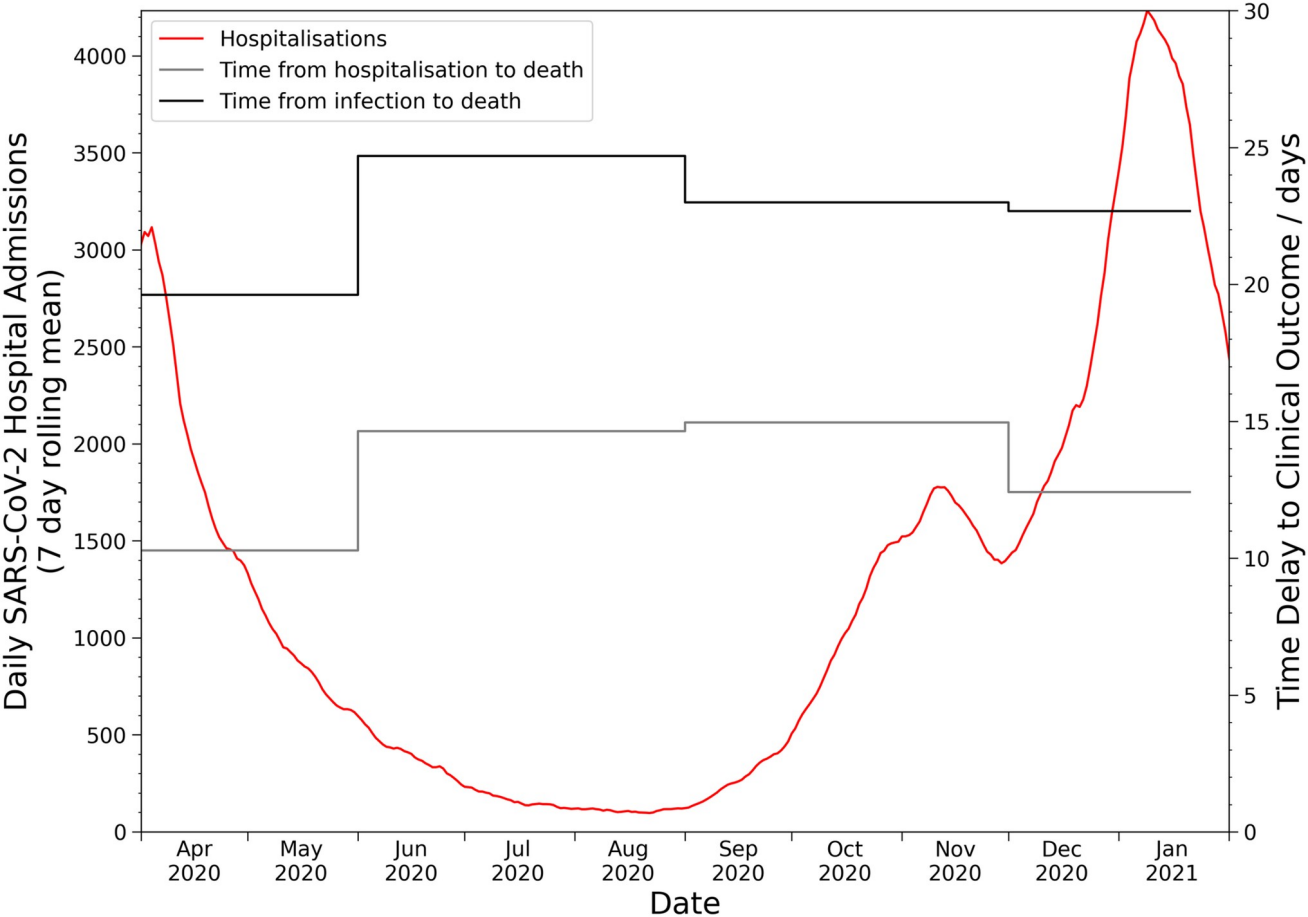
Line graph of the relationship between healthcare pressure, as measured by daily hospital admissions, and the mean modelled clinical time delays.

As seen in Fig 2, there was found to be statistically significant variation between the defined periods. This is particularly apparent in Table 4 where we observe that during the first wave of SARS-CoV-2 the mean time from infection to death is 19.6±0.2 days (95% interval: 5.6, 50.0) and that in the summer period that followed, this rises to 24.7±1.4 days (95% interval: 5.8, 69.8). There has been a substantial change in testing volume and strategy over the timeline of the pandemic impacting the complete capture of COVID-19 deaths and hospitalisations, which will be particularly significant for the January to March 2020 period. This may have had the impact of selection bias at the start of the pandemic albeit the impact of this is thought to be small due to prioritisation of testing for individuals that required clinical care. The summer period is very striking in Fig 2 by the long right tail for all three categories, which could be indicative of a change in patient clinical management as intensive care clinicians found that sustaining patients that were considered extremely critical for longer could result in a higher survival rate [15]. Moreover, the survival rate for patients will have been positively impacted by the endorsement in the UK of dexamethasone [16] use on the 13 November 2020 [17], the more widespread use of individualised lung protective ventilator strategies [18], and the support for proning [19] by the Intensive Care Society [20] in April 2020. High prevalence of SARS-CoV-2 has palpably impacted the healthcare system’s ability to manage the volume of patients [21], which has been a conspicuous impetus behind temporal fluctuations in the clinical time delay distributions, as seen in Fig 3. However, in periods of higher prevalence we may also see a compositional shift towards more severe patients being admitted, which could be seen as an adaptive response to increasing pressure on the healthcare system; nonetheless, this should not have an impact upon the time delay distributions for mortalities. This can be further seen in Table 3 where during the first period hospitalisation to death was 10.3±0.1 days (95% interval: 0.4, 34.9), while an increase was seen in the low prevalence summer to 14.6±0.3 days (95% interval: 0.4, 53.3). This association between an increase in prevalence and a decrease in the time delay to a clinical outcome can be seen across the pandemic in Fig 2. It is perhaps the best early indicator that a healthcare system is under stress and that intervention may be required to allow hospitals to decompress [22]. Table 4 also illustrates that this trend has continued until the most recent period with a mean of 17.4±0.3 (95% interval: 7.7, 33.9) and 22.7±0.4 (95% interval: 13.1, 36.6) for the right truncated model. The results from Fig 2 and Fig A4 in S1 Appendix illustrate that the definitional change of a COVID-19 mortality to be within 28 days of the first laboratory-confirmed positive test [23] does not capture the full distribution of deaths. Analysis of all deaths with confirmed diagnoses of COVID-19 early in the pandemic by Public Health England [24] found that 88% of deaths were within 28 days and 96% were within 60 days of positive COVID-19 test, with 54% of those excluded by the 28 day limit found to have COVID-19 on their death certificate. Moreover, as the results in this study indicate, the mean time to death is longer during times of low prevalence, which leads this categorisation to be more unsuitable. We did not observe a significant impact in the clinical time delay distributions from the growth in the B.1.1.7 variant in December 2020. The changes observed would have been otherwise expected from an increase in overall prevalence. For the study period, it was too early to observe the effects from vaccination campaigns and the concerning B.1.351, B.1.617, and B.1.617.2 variants had a prevalence that would be too low to impact overall time delay trends. Further research should be conducted to understand how vaccination and novel variants affect time delay dynamics.

Corroborating previous literature [5, 6] we find the time from infection to death for SARS-CoV-2 is similar to SARS [25] although a shorter period to peak infectivity is now clear for SARS-CoV-2 [26]. We find that the decrease seen in time from illness onset to hospital admission observed during the SARS outbreak of 2003, thought to be reflective of contact tracing, has not been observed in the SARS-CoV-2 outbreak in the UK. Table 2 illustrates how the time from infection to hospitalisation slightly increased from 8.0±0.1 days (95% interval: 2.7, 18.5) in the first wave to 9.7±0.3 days (95% interval: 4.1, 19.6) at the end of the second wave.

The time from infection to hospitalisation between genders shows a statistically significant difference with males showing a longer modelled mean time of 8.6±0.1 days (95% interval: 2.9, 20.0) relative to 7.9±0.1 days (95% interval: 2.8, 18.0) for females. This difference is not found between genders for the time delay distribution of hospitalisation to death. This is likely related to the well documented epidemiological phenomenon that males have a tendency towards delayed medical help seeking [27]. Galasso et al. (2020) illustrated across eight countries that males are overall likely to be less compliant with NPIs and treat the dangers of COVID-19 with less gravity. The greater fatality rate of males from COVID-19 [28] is a combination of biological, psychosocial, and behavioural causal factors; nonetheless, this delay in seeking out medical attention may be a contributory factor to increasing their overall IFR.

We can observe the differences between age groups in Fig 1. It illustrates that the 40–49 age group have the longest time from infection to death with a mean of 26.5±1.1 days (95% interval: 7.3, 69.3) while the shortest period was found for the 80–89 age group with 17.6±0.2 days (95% interval: 5.3, 44.0). The distribution of the time delays to a clinical outcome seen in Fig 1 illustrates that the youngest and oldest age groups have the shortest time delays, which is revealing of the predominantly more vulnerable nature of the younger adults in 20–39 age bands that require either clinical intervention or have a severe reaction to SARS-CoV-2 infection that results in a mortality.

## Conclusion

We illustrate that evaluating the variation in the time delay temporal changes is key to informing public health policy and that this should not be regarded as a static metric but rather something that, thus far, has been inherently a by-product of extrinsic pressure. By monitoring these changes it will aid in the calibration of quarantine periods, the calculation of fatality rates, and help in unpacking the extent of transmission. This should be monitored closely in response to new variants of concern and further work should aim to understand their time delay dynamics. Moreover, we also recommend further analysis to assess the impact of vaccination campaigns on these trends. The paradigms seen by gender are not unexpected but should help to inform public policy on how to shape the message around when to seek medical attention. Finally, we propose that fluctuations in the modelled mean time from hospitalisation to death can be used as a proxy indicator of healthcare strain and that an intervention is required that may help to preclude avoidable morbidity and mortality. The main limitation of this study is that we can only infer from the wider context any causal impact on the clinical time delay distributions.

## Supporting information

S1 Appendix Category B results and lognormal.stan program listing. (ZIP)
