# Supplementary material for: Understanding an evolving pandemic: An analysis of the clinical time delay distributions of COVID-19 in the United Kingdom
Source: PLoS One. 2021 Oct 20;16(10):e0257978. doi: 10.1371/journal.pone.0257978 (PMC8528322; doi:10.1371/journal.pone.0257978)
Supplement: S1 Appendix — (ZIP) [file pone.0257978.s001.zip › S1_Appendix.pdf]

Table A1. Results for the time from infection to hospitalisation, segmented by hospitalisation date.

Period	N	Model	Mean	SD	α	β	LOOIC	% ($k \leq 0.7$)
01Jan2020 to 31May2020	4287	Lognormal	7.86 (7.75, 7.98)	3.92 (3.80, 4.05)	1.95 (1.94, 1.97)	0.47 (0.46, 0.48)	23483	98
		Gamma	7.97 (7.86, 8.08)	3.74 (3.65, 3.83)	4.55 (4.34, 4.77)	0.57 (0.54, 0.60)	23708	98
		Weibull	8.05 (7.93, 8.17)	3.96 (3.88, 4.05)	2.14 (2.09, 2.19)	9.09 (8.95, 9.22)	24076	94
01Jun2020 to 31Aug2020	149	Lognormal	10.69 (9.61, 11.91)	8.29 (6.83, 10.06)	2.13 (2.03, 2.24)	0.68 (0.62, 0.76)	969	100
		Gamma	11.54 (10.39, 12.80)	8.50 (7.43, 9.76)	1.86 (1.51, 2.25)	0.16 (0.13, 0.20)	1010	100
		Weibull	11.68 (10.38, 13.10)	9.63 (8.44, 11.04)	1.22 (1.10, 1.35)	12.46 (10.97, 14.04)	1024	100
01Sep2020 to 30Nov2020	1020	Lognormal	9.54 (9.27, 9.80)	4.31 (4.04, 4.59)	2.16 (2.13, 2.19)	0.43 (0.41, 0.45)	5867	95
		Gamma	9.58 (9.34, 9.84)	4.17 (3.96, 4.38)	5.30 (4.80, 5.82)	0.55 (0.50, 0.61)	5900	98
		Weibull	9.62 (9.37, 9.89)	4.41 (4.23, 4.60)	2.32 (2.21, 2.43)	10.86 (10.58, 11.16)	5972	94
01Dec2020 to 20Jan2021	537	Lognormal	9.83 (9.41, 10.26)	5.21 (4.76, 5.71)	2.16 (2.12, 2.21)	0.50 (0.46, 0.53)	3205	99
		Gamma	9.95 (9.54, 10.37)	5.06 (4.72, 5.42)	3.89 (3.43, 4.38)	0.39 (0.34, 0.44)	3245	99
		Weibull	10.00 (9.56, 10.43)	5.51 (5.20, 5.84)	1.89 (1.78, 2.00)	11.27 (10.76, 11.76)	3304	98

90% credible intervals are quoted.

Table A2. Results for the time from hospitalisation to death, segmented by date of death.

Period	N	Model	Mean	SD	α	β	LOOIC	% ($k \leq 0.7$)
01Jan2020 to 31May2020	27398	Lognormal	10.44 (10.31, 10.57)	13.39 (13.11, 13.69)	1.86 (1.85, 1.87)	0.99 (0.98, 0.99)	180569	95
		Gamma	9.69 (9.60, 9.77)	8.85 (8.75, 8.96)	1.20 (1.18, 1.22)	0.12 (0.12, 0.13)	178864	100
		Weibull	9.68 (9.60, 9.77)	8.68 (8.58, 8.78)	1.12 (1.11, 1.13)	10.09 (9.99, 10.18)	178807	100
01Jun2020 to 31Aug2020	2662	Lognormal	18.47 (17.78, 19.20)	23.43 (21.89, 25.10)	2.44 (2.40, 2.47)	0.98 (0.96, 1.00)	20560	98
		Gamma	16.80 (16.36, 17.25)	14.44 (13.98, 14.94)	1.35 (1.30, 1.41)	0.08 (0.08, 0.08)	20229	100
		Weibull	16.78 (16.35, 17.23)	13.83 (13.36, 14.33)	1.22 (1.19, 1.25)	17.91 (17.43, 18.41)	20205	100
01Sep2020 to 30Nov2020	12285	Lognormal	13.28 (13.06, 13.50)	15.96 (15.49, 16.44)	2.14 (2.12, 2.15)	0.95 (0.93, 0.96)	86747	96
		Gamma	12.20 (12.05, 12.36)	10.32 (10.15, 10.49)	1.40 (1.37, 1.43)	0.11 (0.11, 0.12)	85386	100
		Weibull	12.18 (12.03, 12.33)	9.82 (9.67, 9.98)	1.25 (1.23, 1.26)	13.07 (12.90, 13.24)	85244	100
01Dec2020 to 20Jan2021	21499	Lognormal	13.22 (13.06, 13.38)	16.02 (15.66, 16.38)	2.13 (2.12, 2.14)	0.95 (0.94, 0.96)	151638	96
		Gamma	12.11 (12.00, 12.23)	10.29 (10.16, 10.41)	1.39 (1.36, 1.41)	0.11 (0.11, 0.12)	149173	100
		Weibull	12.09 (11.98, 12.20)	9.77 (9.66, 9.89)	1.24 (1.23, 1.26)	12.96 (12.84, 13.09)	148908	100

90% credible intervals are quoted.

Table A3. Results for the time from infection to death, segmented by date of death.

Period	N	Model	Mean	SD	α	β	LOOIC	% ($k \leq 0.7$)
01Jan2020 to 31May2020	5023	Lognormal	17.59 (17.38, 17.80)	8.70 (8.47, 8.94)	2.76 (2.75, 2.77)	0.47 (0.46, 0.48)	34927	100
		Gamma	17.61 (17.41, 17.81)	8.23 (8.06, 8.40)	4.58 (4.42, 4.75)	0.26 (0.25, 0.27)	35097	100
		Weibull	17.67 (17.45, 17.89)	8.56 (8.42, 8.71)	2.18 (2.13, 2.22)	19.95 (19.71, 20.20)	35493	99
01Jun2020 to 31Aug2020	404	Lognormal	45.06 (42.06, 48.41)	39.47 (34.82, 44.78)	3.52 (3.46, 3.59)	0.75 (0.71, 0.80)	3790	100
		Gamma	44.09 (41.61, 46.67)	31.66 (29.31, 34.19)	1.95 (1.74, 2.17)	0.04 (0.04, 0.05)	3803	100
		Weibull	44.45 (41.94, 47.05)	31.60 (29.38, 34.11)	1.43 (1.34, 1.52)	48.89 (46.01, 51.92)	3815	100
01Sep2020 to 30Nov2020	1112	Lognormal	25.00 (24.15, 25.86)	18.29 (17.16, 19.56)	3.00 (2.97, 3.04)	0.65 (0.63, 0.68)	8947	100
		Gamma	26.57 (25.63, 27.52)	19.17 (18.28, 20.12)	1.92 (1.80, 2.05)	0.07 (0.07, 0.08)	9309	100
		Weibull	26.90 (25.80, 28.06)	22.30 (21.23, 23.44)	1.21 (1.17, 1.25)	28.67 (27.42, 29.96)	9452	100
01Dec2020 to 20Jan2021	987	Lognormal	23.73 (22.98, 24.50)	14.39 (13.50, 15.37)	3.01 (2.98, 3.04)	0.56 (0.54, 0.58)	7662	100
		Gamma	24.06 (23.32, 24.79)	13.87 (13.25, 14.54)	3.01 (2.79, 3.25)	0.13 (0.12, 0.14)	7789	100
		Weibull	24.24 (23.41, 25.09)	15.61 (14.98, 16.27)	1.59 (1.53, 1.65)	27.02 (26.06, 27.99)	7937	100

The modelled parameters for 1 June to 31 August should be treated with caution because they appear to be skewed by infections that occurred in the first wave. 90% credible intervals are quoted.

Fig A4. Violin plots of the best fit modelled distributions of times to clinical outcome over the course of the pandemic for category B. The quartiles for each distribution are shown as dashed lines and the solid line corresponds to the mean. In this chart, the data are segmented in time by the latter of the two events: hospitalisation date for infection to hospitalisation and date of death for both hospitalisation to death and infection to death. For all three quantities, the mean time was lowest in the first wave, and there was a marked increase over the summer months. The modelled distribution for infection to death from 1 June to 31 August should be treated with caution because it appears to be skewed by infections that occurred in the first wave.

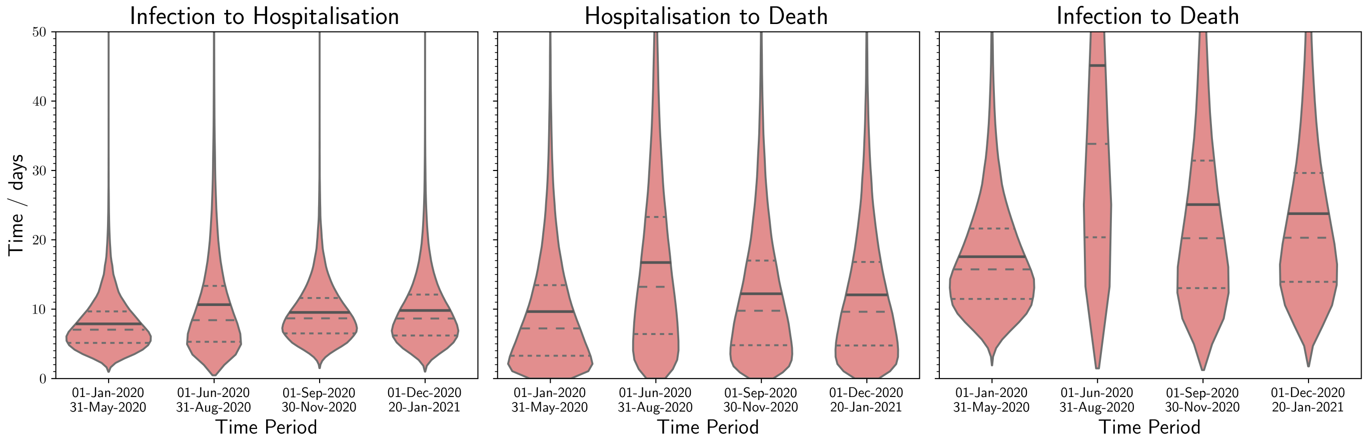

lognormal.stan: the Stan program for the lognormal model without right truncation.

```
// distribution: lognormal
// truncation:   no

data {
  int<lower = 0> N;                  // number of records
  vector<lower = 0>[N] a_minus;      // lower limit of event A
  vector<lower = 0>[N] a_plus;       // upper limit of event A
  vector<lower = 0>[N] b_minus;      // lower limit of event B
  vector<lower = 0>[N] b_plus;       // upper limit of event B
```

```
  int<lower = 0, upper = 1> incubation; // inclusion of time from O to A (incubation period)
}

parameters {
  real logmean;                                // natural log of the mean
  real logsd;                                  // natural log of the standard deviation
  vector<lower = 0, upper = 1>[N] a_window; // where time a lies in the event A window
  vector<lower = 0, upper = 1>[N] b_window; // where time b lies in the event B window
  vector<lower = 0>[N] t0;                     // time from O to A
}

transformed parameters {
  real<lower = 0> beta = sqrt(log1p_exp(2 * (logsd - logmean)));
  real alpha = logmean - (beta^2)/2;

  vector<lower = min(a_minus), upper = max(a_plus)>[N] a;
  vector<lower = min(b_minus), upper = max(b_plus)>[N] b;
  vector[N] ub;

  b = b_minus + (b_plus - b_minus) .* b_window;

  for (n in 1:N)
    ub[n] = min([a_plus[n], b[n]]');
  a = a_minus + (ub - a_minus) .* a_window;
}

model {
  logmean ~ std_normal();
  logsd ~ std_normal();

  if (incubation)
    t0 ~ lognormal(1.63, 0.5);
  else
    t0 ~ normal(0, 1e-10);

  target += lognormal_lpdf((b - a + t0) | alpha, beta);
}

generated quantities {
  real<lower = 0> mean_ = exp(alpha + (beta^2)/2);
  real<lower = 0> sd_ = sqrt((exp(beta^2) - 1) * exp(2*alpha + beta^2));

  vector[N] log_likelihood;
  for (n in 1:N)
    log_likelihood[n] = lognormal_lpdf(b[n] - a[n] + t0[n] | alpha, beta);
}
```
